# Supplementary material for: Development of a Novel Anti-EpCAM Monoclonal Antibody for Various Applications
Source: Antibodies (Basel). 2022 Jun 8;11(2):41. doi: 10.3390/antib11020041 (PMC9220218; doi:10.3390/antib11020041)
Supplement: Supplementary file 1 [file antibodies-11-00041-s001.zip › antibodies-1677625-supplementary.pdf]

antibodies

MDPI

Supplemental materials

A

EpCAM_24_43	QEESVSENYKLAVNSFVNNN
EpCAM_34_53	LAVNSFVNNNRQSQSTSVGA
EpCAM_44_63	RQSQSTSVGAQNTVISSKLA
EpCAM_54_73	QNTVISSKLAAKSLVMKAEM
EpCAM_64_83	AKSLVMKAEMNGSKLGRRAK
EpCAM_74_93	NGSKLGRRAKPEGALQNNDG
EpCAM_84_103	PEGALQNNDGLYDPDSDESG
EpCAM_94_113	LYDPDSDESGLFKAKQSNGT
EpCAM_104_123	LFKAKQSNGTSTSWSVNTAG
EpCAM_114_133	STSWSVNTAGVRRTDKDTEI
EpCAM_124_143	VRRTDKDTEITSSERVRTYW
EpCAM_134_153	TSSERVRTYWIIIELKHKAR
EpCAM_144_163	IIIELKHKAREKPYDSKSLR
EpCAM_154_173	EKPYDSKSLRTALQKEITTR
EpCAM_164_183	TALQKEITTRYQLDPKFITS
EpCAM_174_193	YQLDPKFITSILYENNVITI
EpCAM_184_203	ILYENNVITIDLVQNSSQKT
EpCAM_194_213	DLVQNSSQKTQNDVDIADVA
EpCAM_204_223	QNDVDIADVAYYFEKDVKGE
EpCAM_214_233	YYFEKDVKGESLFHSKKMDL
EpCAM_224_243	SLFHSKKMDLTVNGEQLDLD
EpCAM_234_253	TVNGEQLDLDPGQTLIYYVD
EpCAM_244_262	PGQTLIYYVDEKAPEFSMQ-

B

	1	2	3	4	5	6	7	8	9
A	24-43	24-43	24-43	104-123	104-123	104-123	184-203	184-203	184-203
B	34-53	34-53	34-53	114-133	114-133	114-133	194-213	194-213	194-213
C	44-63	44-63	44-63	124-143	124-143	124-143	204-223	204-223	204-223
D	54-73	54-73	54-73	134-153	134-153	134-153	214-233	214-233	214-233
E	64-83	64-83	64-83	144-163	144-163	144-163	224-243	224-243	224-243
F	74-93	74-93	74-93	154-173	154-173	154-173	234-253	234-253	234-253
G	84-103	84-103	84-103	164-183	164-183	164-183	244-262	244-262	244-262
H	94-113	94-113	94-113	174-193	174-193	174-193	P.C.	P.C.	P.C.

	1	2	3	4	5	6	7	8	9
A	0.051	0.051	0.052	0.049	0.048	0.047	0.055	0.051	0.056
B	0.044	0.039	0.04	0.042	0.038	0.039	0.038	0.039	0.042
C	0.041	0.037	0.038	0.04	0.038	0.04	0.04	0.041	0.043
D	0.04	0.036	0.036	0.049	0.048	0.05	0.039	0.042	0.042
E	0.043	0.039	0.038	0.038	0.036	0.038	0.039	0.041	0.04
F	0.042	0.038	0.038	0.039	0.04	0.039	0.044	0.045	0.045
G	0.045	0.045	0.039	0.042	0.041	0.041	0.043	0.044	0.047
H	0.05	0.042	0.042	0.044	0.046	0.046	0.725	0.712	0.737

positive control

C

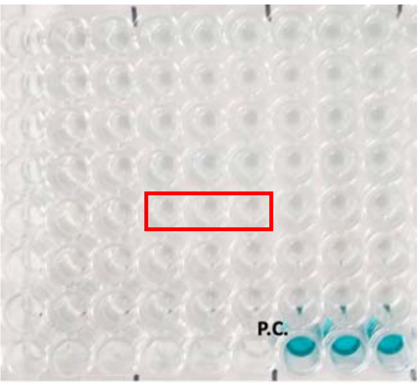

positive control

Figure S1. Determination of the recEpMab-37 epitope for EpCAM by ELISA using synthesized peptides of EpCAM extracellular domain. (A) Synthesized peptides of EpCAM extracellular domain. (B, C) The peptides (10 μg/mL) were immobilized on immunoplates as indicated. The plates were incubated with recEpMab-37 (1 μg/mL), followed by incubation with peroxidase-conjugated anti-mouse immunoglobulins. Enzymatic reactions were performed using the ELISA POD Substrate. Optical density was measured at 655 nm. Recombinant EpCAM ectodomain was used as a positive control. The Red square indicates the candidate region of recEpMab-37 epitope determined by flow cytometry (Fig. 4 in the main text). ELISA, enzyme-linked immunosorbent assay .
